# One out of Four: HspL but No Other Small Heat Shock Protein of *Agrobacterium tumefaciens* Acts as Efficient Virulence-Promoting VirB8 Chaperone

**DOI:** 10.1371/journal.pone.0049685

**Published:** 2012-11-21

**Authors:** Yun-Long Tsai, Yin-Ru Chiang, Chih-Feng Wu, Franz Narberhaus, Erh-Min Lai

**Affiliations:** 1 Institute of Plant and Microbial Biology, Academia Sinica, Taipei, Taiwan; 2 Lehrstuhl für Biologie der Mikroorganismen, Ruhr-Universität Bochum, Bochum, Germany; University of Wisconsin-Milwaukee, United States of America

## Abstract

Alpha-crystallin-type small heat shock proteins (sHsps) are ubiquitously distributed in most eukaryotes and prokaryotes. Four sHsp genes named *hspL*, *hspC*, *hspAT1*, and *hspAT2* were identified in *Agrobacterium tumefaciens*, a plant pathogenic bacterium capable of unique interkingdom DNA transfer via type IV secretion system (T4SS). HspL is highly expressed in virulence-induced growth condition and functions as a VirB8 chaperone to promote T4SS-mediated DNA transfer. Here, we used genetic and biochemical approaches to investigate the involvement of the other three sHsps in T4SS and discovered the molecular basis underlying the dominant function of HspL in promoting T4SS function. While single deletion of *hspL* but no other sHsp gene reduced T4SS-mediated DNA transfer and tumorigenesis efficiency, additional deletion of other sHsp genes in the *hspL* deletion background caused synergistic effects in the virulence phenotypes. This is correlated with the high induction of *hspL* and only modest increase of *hspC*, *hspAT1*, and *hspAT2* at their mRNA and protein abundance in virulence-induced growth condition. Interestingly, overexpression of any single sHsp gene alone in the quadruple mutant caused increased T4SS-mediated DNA transfer and tumorigenesis. Thermal aggregation protecting assays *in vitro* indicated that all four sHsps exhibit chaperone activity for the model substrate citrate synthase but only HspL functions as efficient chaperone for VirB8. The higher VirB8 chaperone activity of HspL was also demonstrated *in vivo,* in which lower amounts of HspL than other sHsps were sufficient in maintaining VirB8 homeostasis in *A. tumefaciens.* Domain swapping between HspL and HspAT2 indicated that N-terminal, central alpha-crystallin, and C-terminal domains of HspL all contribute to HspL function as an efficient VirB8 chaperone. Taken together, we suggest that the dominant role of HspL in promoting T4SS function is based on its higher expression in virulence-induced condition and its more efficient VirB8 chaperone activity as compared to other sHsps.

## Introduction

Alpha-crystallin type small heat-shock proteins (sHsps) are ubiquitous proteins of low molecular-mass chaperones consisting of a conserved alpha-crystallin domain [1]. In general, sHsps function to prevent irreversible protein aggregation in an ATP-independent manner by partially binding denatured proteins and facilitating the subsequent refolding by ATP-dependent chaperones [2]. The chaperone activity is generally determined by their ability in protecting thermolabile artificial model substrates; e.g. citrate synthase (CS), firefly luciferase (Luc), or malate dehydrogenase (MDH); from aggregation *in vitro* [3], [4]. sHsps show extensive sequence variations but are structurally conserved with their native state as oligomers in the range of 12 to >48 subunits [5]. sHsps contain a highly conserved α-crystallin domain flanked by a variable N-terminal region and a C-terminal extension [1]. Each of the three domains are required and play both distinct and cooperative roles in conferring chaperone activity [1], [3], [6]–[9]. The N-terminal arm plays a major role in substrate recognition and also affects oligomerization of the chaperone [3], [5], [10] whereas the α-crystallin domain is important for dimerization and oligomerization [11], [12]. The function of the C-terminal extension is less understood but some evidence suggests its role for integrity of sHsp oligomers [5].

Several bacterial sHsp genes are massively induced by heat shock or non-heat shock stresses [1]. Notably, several soil bacteria belonging to the *Rhizobiaceae* possess multiple sHsps. However, it remains unknown how and why these rhizobia evolved or acquired multiple sHsps, which presumably function to protect cells under various stresses. It was suggested that these redundant copies may rapidly elevate the amounts of protective proteins under stress conditions [13], [14]. In addition, different sHsps may form hetero-oligomers, which might provide flexibility in response to multiple stresses [15].

*Agrobacterium tumefaciens* is the causal agent of crown gall disease with the unique ability to transfer its DNA into plant cells via a type IV secretion system (T4SS) [16], [17]. The T4SS consists of a VirD4 coupling protein and 11 VirB proteins (VirB1 to VirB11) [18]. VirB8 functions as an inner membrane dimer and may cooperate with VirB6 to serve as a inner membrane base complex in connecting the VirB7/VirB9/VirB10 core channel across the double membrane [19]–[22]. In *A. tumefaciens*, four sHsp genes (*hspC*, *hspL*, *hspAT1*, and *hspAT2*) have been identified and the latter three genes are transcriptionally induced by heat shock [23]. Interestingly, HspL was discovered by our previous proteomics study, in which it was highly induced at room temperature by acetosyringone (AS), a potent virulence gene inducer [24]. AS-induced HspL accumulation is regulated at both transcriptional and posttranslational levels in a VirB-dependent manner [25] and functions as a VirB8 chaperone in promoting T4SS-mediated DNA transfer and tumorigenesis [26]. In contrast, the functions of other three sHsp genes remain unknown.

In this study, we investigated the biochemical and biological functions of the four *Agrobacterium* sHsps. We discovered that the dominant role of HspL over HspC, HspAT1, and HspAT2 in promoting T4SS-mediated DNA transfer and virulence is contributed by both expression levels and biochemical activity as the VirB8 chaperone.

## Results

### Impact of Single and Multiple Deletions of sHsp Genes in T4SS-mediated DNA Transfer and Virulence of *A. tumefaciens*

**Figure 1:**
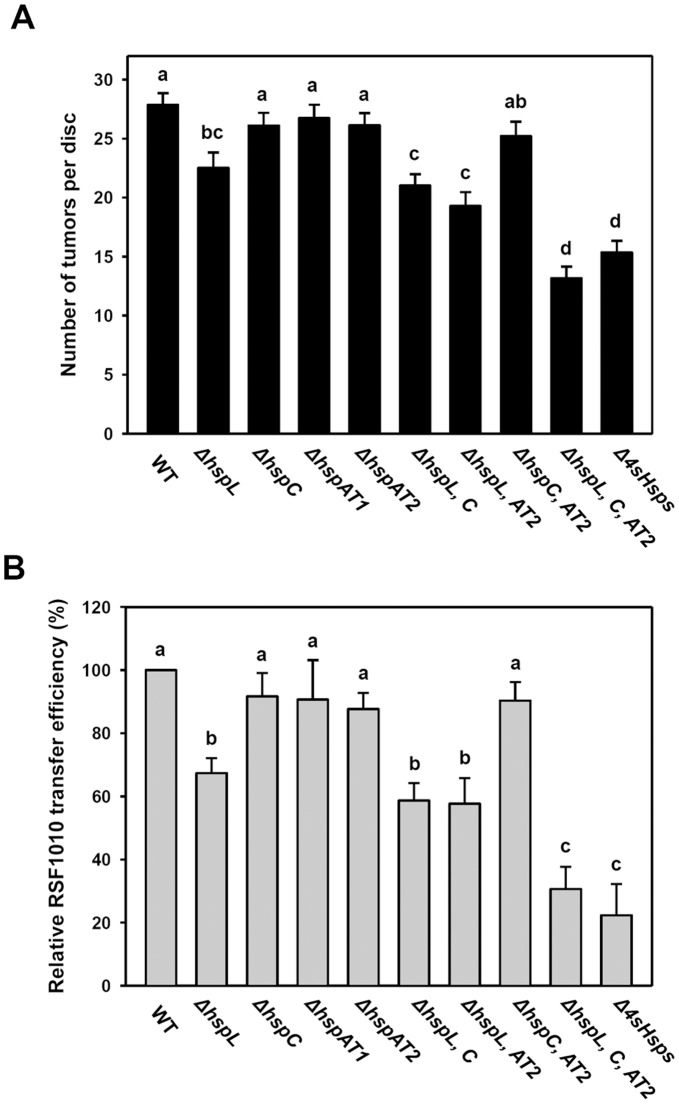
Tumorigenesis and RSF1010 transfer efficiency assays of single and multiple sHsp deletion mutants. (A) Tumorigenesis efficiency is presented as number of tumor per disc averaged from more than 60 potato discs, with standard errors. Three independent experiments were carried out with similar results with the representative result shown. (B) The RSF1010 transfer efficiency was evaluated as number of transconjugants per input donor. Average values for relative RSF1010 transfer efficiency from three independent experiments are shown with standard errors, in which the efficiency of wild type strain NT1RE(pJK270) (WT) was set at 100% and that of other strains is shown relative to that of NT1RE(pJK270). Means annotated with the same letter (a-d) are not significantly different; those with different letters are significantly different (*P*<0.05) according to Duncan’s multiple range test. The absolute RSF1010 transfer efficiency and the numbers of transconjugants and input donors obtained from three independent experiments were shown in Table 1.

Among four sHsp genes (*hspL, hspC, hspAT1, hspAT2*) encoded in the *A. tumefaciens* strain C58 genome, we previously discovered that *hspL* is a virulence factor in promoting T4SS-mediated DNA transfer and virulence [25]. However, it remains unknown whether *hspC, hspAT1,* or *hspAT2* play a role in the function of T4SS in *A. tumefaciens.* Thus, we generated single deletion mutants of *hspC, hspAT1,* and *hspAT2* and determined their phenotypes in T4SS-mediated RSF1010 transfer between agrobacteria and tumorigenesis efficiency on potato tuber discs. Similar to our previous findings [25], the Δ*hspL* mutant caused modest but significant reduction in both tumorigenesis and RSF1010 transfer efficiency (Figure 1A and 1B). However, no phenotypes could be detected from each of the Δ*hspC,* Δ*hspAT1,* or Δ*hspAT2* single deletion mutants or even the Δ*hspC* Δ*hspAT2* double mutant. Interestingly, additional deletion of other sHsp genes in Δ*hspL,* however, caused synergistic effects in abrogating T4SS functions as evidenced from further reduction in both tumorigenesis and RSF1010 transfer efficiencies in these triple (Δ*L, C*, *AT2*) and quadruple mutants (Δ*L, C*, *AT1, AT2,* also named as Δ*4sHsps*) (Figure 1A, 1B, Table 1). Taken together, these results suggest that while HspL remains as the most important sHsp in promoting T4SS-mediated DNA transfer and virulence, other sHsps may also contribute to T4SS function but to lesser extent in *A. tumefaciens*.

**Table 1:** Mobilization efficiency of RSF1010 derivative PML122*Δkm*::Tc^R^ between *Agrobacterium* strains.

Donor strain	Mean no. of transconjugants ± SD	Mean no. of inputdonors ± SD (× 10^7^)	Mean no. ± SD oftransconjugants/input donor (x 10^−6^)	Relative transfer efficiency (%) to that of NT1RE(pJK270)
WT: NT1RE (pJK270)	44±13	(1.2±0.3)	3.6±0.2	100%
*ΔhspL*	32±10	1.3±0.4	2.4±0.1	67%
*ΔhspC*	40±8	1.2±0.3	3.3±0.2	92%
*ΔhspAT1*	40±10	1.3±0.4	3.2±0.5	91%
*ΔhspAT2*	40±11	1.3±0.3	3.1±0.3	88%
*ΔhspL*, *C*	29±9	1.4±0.3	2.1±0.4	59%
*ΔhspL*, *AT2*	26±7	1.3±0.3	2.1±0.4	58%
*ΔhspC*, *AT2*	42±11	1.3±0.3	3.2±0.3	91%
*ΔhspL*, *C*, *AT2*	13±1	1.3±0.3	1.1±0.2	31%
*Δ4sHsps*	9±3	1.2±0.3	0.8±0.4	22%

### Expression Analysis of Four sHsp Genes

**Figure 2:**
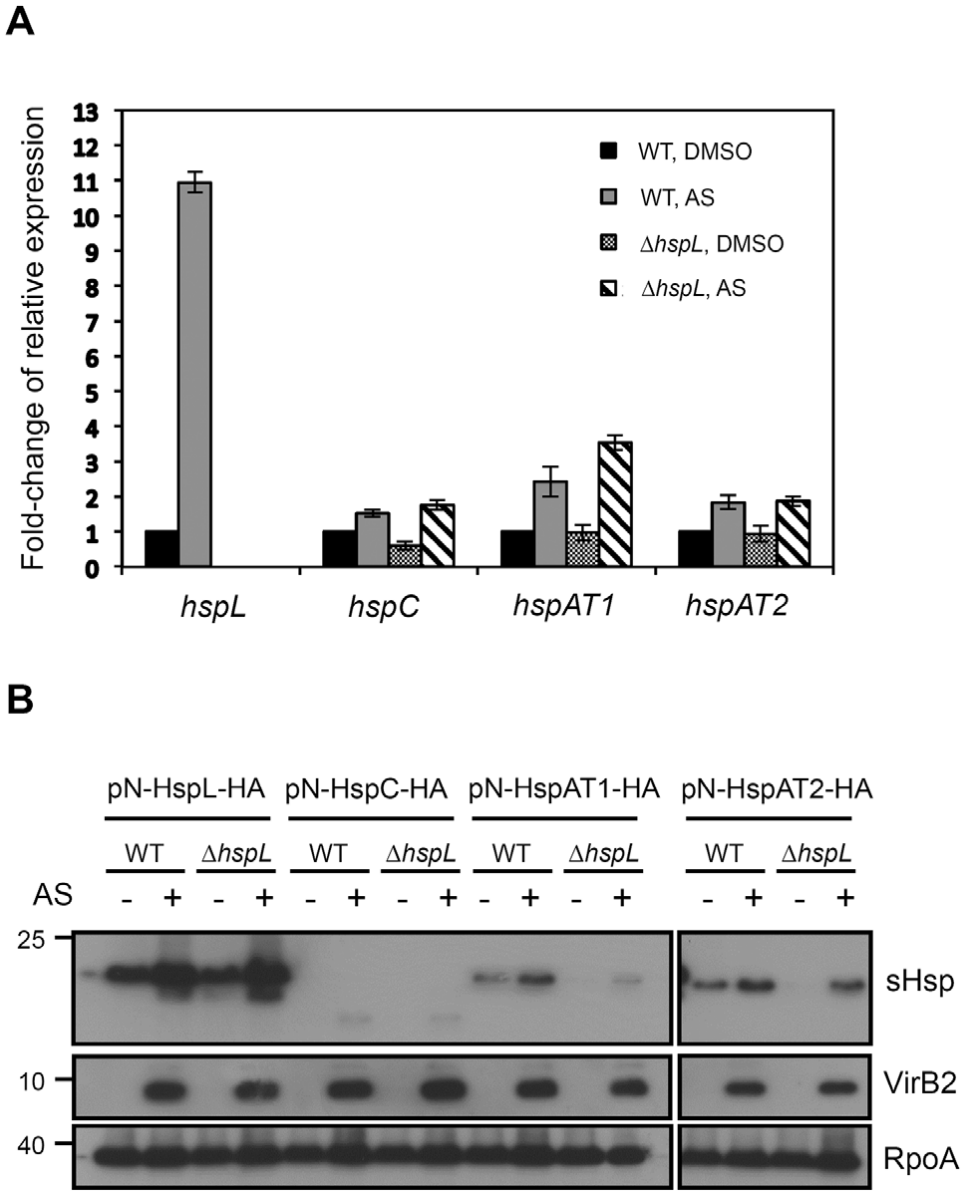
AS-induced mRNA and protein expression of four sHsp genes in *A. tumefaciens*. (A) Quantitative RT-PCR analysis of sHsp mRNA levels in *A. tumefaciens* wild type (WT) strain NT1RE(pJK270) and the *ΔhspL* mutant. Relative expression level is normalized by 16S rRNA as an internal control and the mean values of fold-change relative to the DMSO control of WT. Data are mean with standard deviation (SD) of 2 biological replicates, each of which contains 3 technical replicates. (B) Each of the sHsp genes expressing proteins tagged with HA and driven by its upstream promoter region on plasmid was expressed in wild type (WT) strain NT1RE(pJK270) and the *ΔhspL* mutant. The agrobacterial cells were grown in ABMES medium (pH5.5) at 25°C in the absence (−, DMSO control) or the presence of AS for 16 hrs. The total cell lysates were detected by western blot analysis with specific antibody against HA, VirB2, or RpoA. Numbers on the left are molecular masses of reference proteins in kDa.

Our mutant analysis strongly suggested that the other three *shsp* genes may partially compensate the function of *hspL* in promoting T4SS-mediated DNA transfer and virulence of *A. tumefaciens.* However, it is unknown whether the observed *hspC, hspAT1,* and *hspAT2*-mediated phenotypes are attributed to the levels of expression or biochemical activity. By transcriptional fusion of the promoter to *gfp* (green fluorescence protein), we previously identified that *hspL* but not *hspC, hspAT1,* or *hspAT2* were upregulated by the *vir* gene inducer acetosyringone (AS) [25]. While both mRNA and proteins levels of *hspL* are highly induced by AS [25], the steady state levels of the native mRNA transcripts and protein products of *hspC, hspAT1,* and *hspAT2* under AS-induced condition remain unknown. Thus, we first carried out quantitative RT-PCR (qRT-PCR) to determine their relative mRNA levels when induced by AS. As expected, *hspL* mRNA is massively upregulated by AS in wild type *A. tumefaciens* (WT) (Figure 2A). Interestingly, the mRNA abundance for *hspC, hspAT1,* and *hspAT2* were also modestly induced by AS in both WT and Δ*hspL* mutant as compared to the negative (DMSO) control. To determine whether the mRNA levels reflect the protein levels, each sHsp gene encoding the protein tagged with hemagglutinin (HA) was expressed from its native promoter on plasmid in both WT and Δ*hspL* mutant and detected by anti-HA with western blot analysis. While HspL-HA remained the most abundant AS-induced sHsp, we were able to detect elevated expression of HA-tagged HspC, HspAT1, and HspAT2 in the presence of AS as compared to the control without AS induction at room temperature (Figure 2B). RNA polymerase alpha subunit (RpoA) was used as an internal control and VirB2 protein served a AS-induction control to ensure that the discrepancy of AS-induced sHsp protein levels is not due to the difference of AS induction efficiency in different strains (Figure 2B). These data suggested that HspL is the major sHsp induced by AS but the other three sHsps are also slightly induced by AS in the presence or absence of HspL.

### Overexpression of Single sHsp Gene Alone in *Δ4sHsps* Causes the Increased T4SS-mediated DNA Transfer and Tumorigenesis

**Figure 3:**
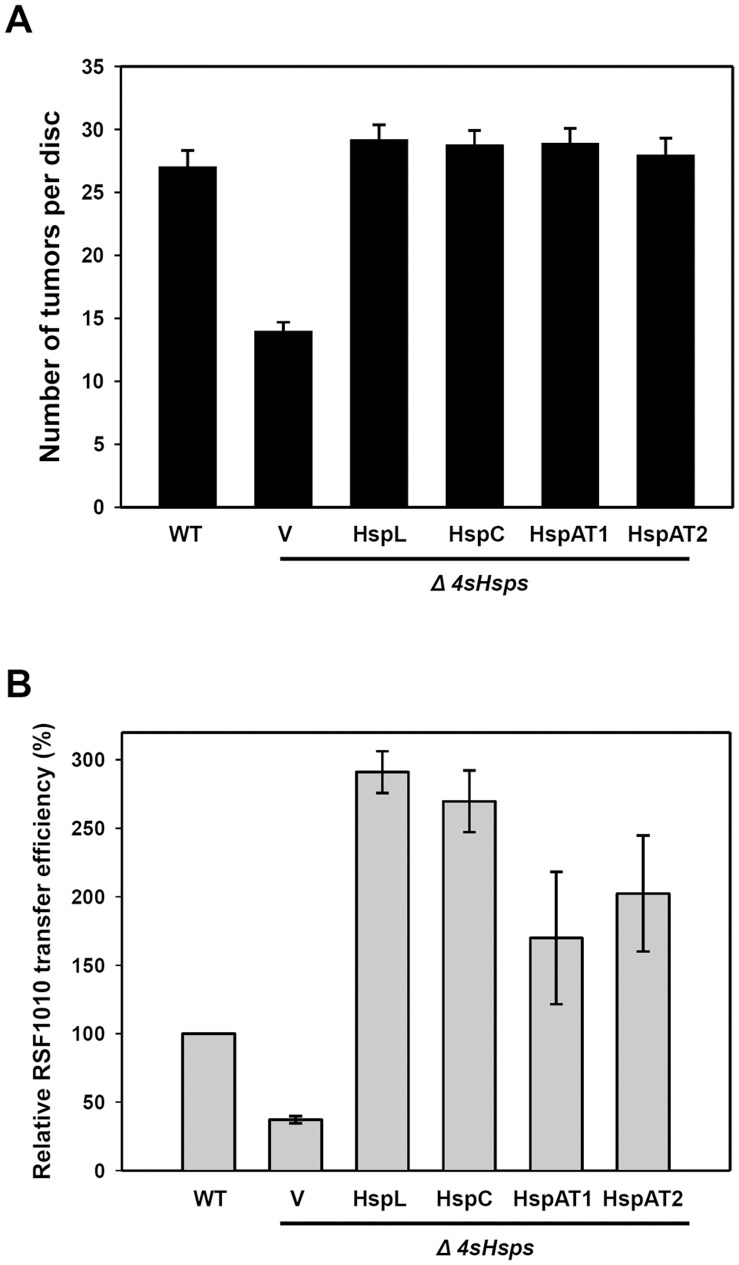
Effect of overproduced sHsp in tumorigenesis and T4SS-mediated DNA transfer. Wild type NT1RE(pJK270) (WT), the quadruple sHsp deletion mutant *Δ4sHsps* harboring pRL662 (V) or pRL662::*hspL* (HspL), pRL662::*hspC* (HspC), pRL662::*hspAT1* (HspAT1), pRL662::*hspAT2* (HspAT2) were assayed for their tumorigenesis efficiency on potato tuber discs and RSF1010 transfer efficiency as described in Figure 1. The absolute RSF1010 transfer efficiencies obtained from each of the three independent experiments were shown in Table 2.

The expression data suggested that the minor role of HspC, HspAT1 and HspAT2 in promoting T4SS function could be due to their modest induction albeit low abundance in virulence-induced growth condition. If this is the case, elevation of HspC, HspAT1, and HspAT2 protein levels to that of HspL should be able to pheno-copy the function of HspL. To test this hypothesis, we overexpressed each of the sHsp genes driven by a constitutively expressed *lac* promoter in the quadruple deletion mutant (Δ*4sHsps*) and determined their effects in T4SS-mediated tumorigenesis and RSF1010 transfer efficiency. Overexpression of each of sHsps in Δ*4sHsps* was able to restore the tumorigenesis efficiency at wild type-like levels (Figure 3A). Interestingly, overexpression of the individual sHsps not only rescued the defect in reduced RSF1010 transfer efficiency of the Δ*4sHsps* mutant but caused significantly higher efficiency than that of wild type (Figure 3B and Table 2). Specifically, overexpression of HspL or HspC resulted in ∼3-fold higher and HspAT1 or HspAT2 caused ∼1.5- to 2-fold higher than that of wild type (Figure 3B). This result suggested that the RSF1010 transfer assay between agrobacteria may provide higher sensitivity to determine T4SS-mediated DNA transfer than quantitative tumor assays on potato tuber discs. Alternatively, different sHsps may exert their differential functions depending on the substrates and recipients the T4SS encounters.

**Table 2:** Effect of overproduced-small heat shock proteins on mobilization efficiency of RS1010 derivative 122*Δkm*::Tc^R^ between *Agrobacterium* strains.

Donor strain	Conjugation frequency (%)^a^		
	Experiment 1	Experiment 2	Experiment 3
WT: NT1RE (pJK270)	7.3×10^−6^ (100%)	3.6×10^−6^ (100%)	6.5×10^−6^ (100%)
NT1RE (pEL1000) : *ΔvirB* operon	5.7×10^−7^ (8%)	2.3×10^−7^ (6%)	6.4×10^−7^ (10%)
*Δ4sHsps* (pRL662)	2.5×10^−6^ (34%)	1.4×10^−6^ (39%)	2.5×10^−6^ (38%)
*Δ4sHsps* (pRL662::*hspL*)	2.1×10^−5^ (287%)	1.0×10^−5^ (278%)	2.0×10^−5^ (308%)
*Δ4sHsps* (pRL662::*hspC*)	2.1×10^−5^ (287%)	8.8×10^−6^ (244%)	1.8×10^−5^ (277%)
*Δ4sHsps* (pRL662::*hspAT1*)	8.7×10^−6^ (119%)	6.3×10^−6^ (175%)	1.4×10^−5^ (215%)
*Δ4sHsps* (pRL662::*hspAT2*)	1.0×10^−5^ (192%)	9.0×10^−6^ (250%)	1.1×10^−5^ (169%)

a expressed as number of transconjugants per input donor.

### All Four sHsps Exhibit Chaperone Activity for Citrate Synthase

**Figure 4:**
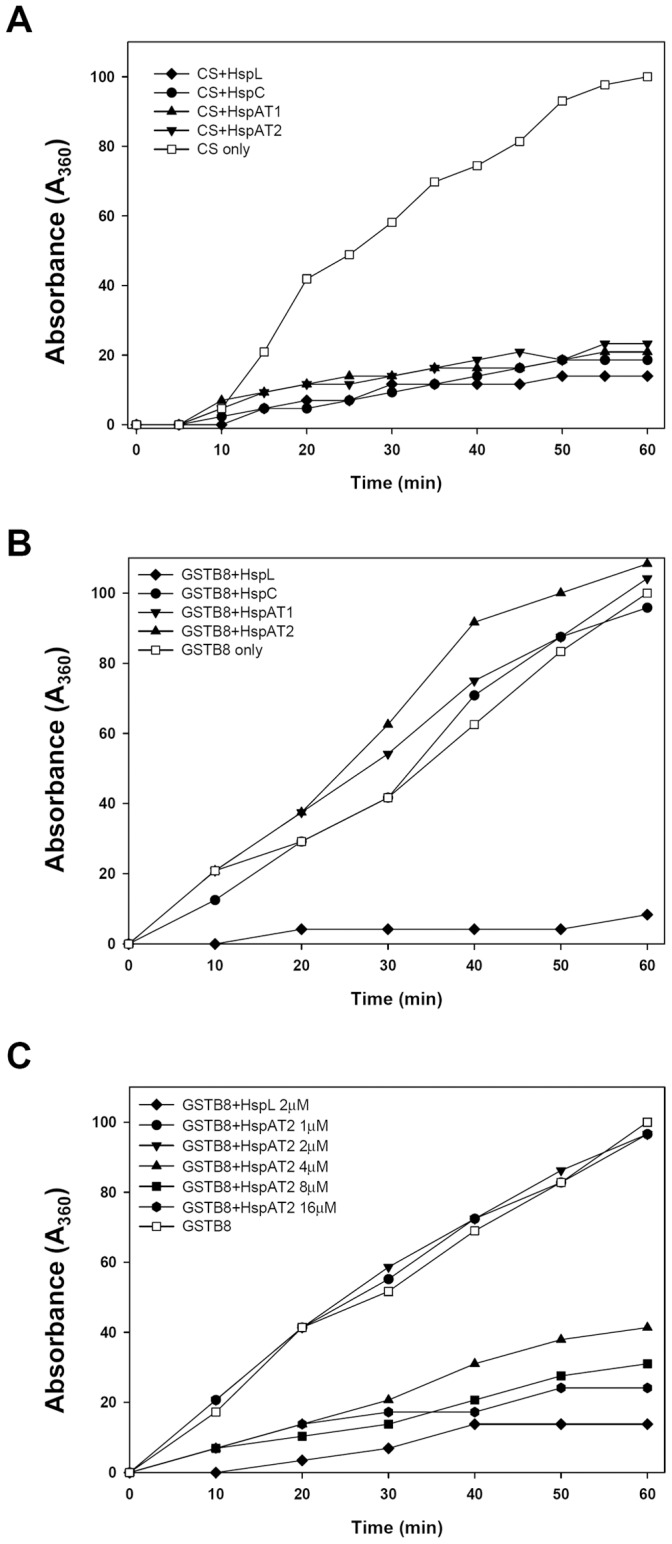
Chaperone activity assay of sHsps. (A) Thermal aggregation protection assays using model substrate CS (600 nM) was carried out at 43°C in the absence (□) or presence of HspL-His_6_ (♦), HspC-His_6_ (•), HspAT1-His_6_ (▴) or HspAT2-His_6_ (▾) at concentration of 1.2 µM respectively. (B) 1 µM GST-VirB8 was used as substrate and was carried out at 50°C in the absence (□) or presence of HspL-His_6_ (♦), HspC-His_6_ (•), HspAT1-His_6_ (▾) or HspAT2-His_6_ (▴) at concentration of 2 µM respectively. (C) 1 µM GST-VirB8 was used as substrate and was carried out at 50°C in the absence (□) or presence of HspAT2-His_6_ with concentration at 1 µM(•), 2 µM(▾), 4 µM(▴), 8 µM(▪) and 16 µM (<$>\raster="rg1"<$>), or of 2 µM HspL-His_6_ (♦). Aggregation was monitored in absorbance at 360 nm and presented as a function of time.

Restoration of T4SS-mediated DNA transfer and tumorigenesis efficiency in Δ*4sHsps* by HspC, HspAT1, and HspAT2 suggested that these three sHsp proteins may function as molecular chaperones like HspL [26]. Thus, we overexpressed and purified all four sHsps tagged with 6xHis in *E. coli* and determined their chaperone activities by a thermal aggregation protection assay [26]. The results show that all four sHsps are equally efficient in protecting the model substrate citrate synthase (CS) from thermal aggregation *in vitro* (Figure 4A). Thus, we concluded that HspC, HspAT1, and HspAT2 are typical sHsps with characteristic chaperone activity. This result also explains their ability in promoting T4SS functions by overexpression in the Δ*4sHsps* mutant (Figure 3 and Table 2).

### HspL Exhibits Higher Chaperone Activity than Other sHsps in Protecting GST-VirB8 from Thermal Aggregation *in vitro*

It is intriguing that overexpression of HspL or HspC conferred higher RSF1010 transfer efficiency than that of HspAT1 or HspAT2 in the Δ*4sHsps* mutant (Figure 3B). This raises a possibility that these sHsps may exhibit different substrate specificities *in vivo*. Thus, we next determined the chaperone activity of all four sHsps using VirB8 fused with glutathione-S-transferase (GST) as a substrate. While HspL was capable of protecting GST-VirB8 from thermal aggregation, no VirB8 chaperone activity for HspC, HspAT1, or HspAT2 could be detected at a molar ratio of Hsp/substrate of 2 (Fig. 4B), the optimal ratio for full chaperone activity of HspL [26].

The structure and biochemical activity of sHsp is highly dynamic in a concentration-dependent manner [1]. Thus, it is possible that the amounts of purified HspC, HspAT1, and HspAT2 used to detect their chaperone activity for GST-VirB8 were not sufficient. Thus, we selected HspAT2, whose ability in promoting RSF1010 transfer efficiency was lower than that of HspL when expressed in the Δ*4sHsps* mutant, to test this possibility. As shown in Figure 4C, HspAT2 was able to protect GST-VirB8 from aggregation with an increased Hsp/substrate molar ratio to 4. The HspAT2 chaperone activity for VirB8-GST was further elevated when the molar ratio was set to 8 or 16, when the activity was close to that of HspL with a molar ratio of 2 (Figure 4C). Thus, we concluded that HspAT2 also possesses chaperone activity for VirB8 but less efficient than HspL. This difference in their biochemical activity is correlated with their *in vivo* function in promoting T4SS-mediated RSF1010 transfer efficiency.

### HspL Possesses Higher Activity than HspAT2 in Maintaining VirB8 Homeostasis *in vivo*

**Figure 5:**
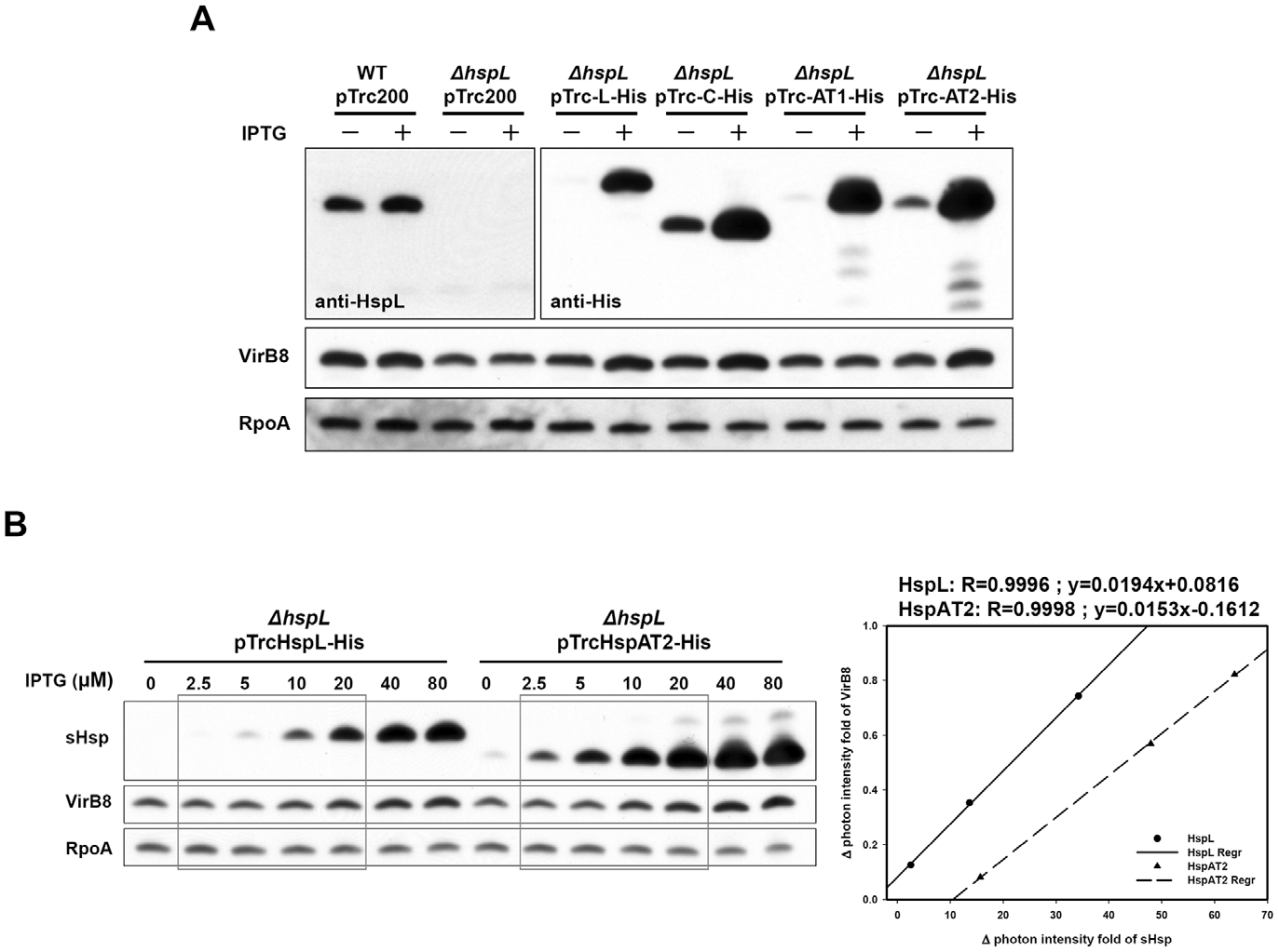
Effects of IPTG-induced His-tagged HspL, HspC, HspAT1 or HspAT2 on VirB8 accumulation. (A) *ΔhspL*(pTrc-HspL-His), *ΔhspL*(pTrc-HspC-His), *ΔhspL*(pTrc-HspAT1-His), and *ΔhspL*(pTrc-HspAT2-His) were grown in AB-MES (pH 5.5) containing 200 µM AS at 28°C with or without 0.4 mM IPTG for 24 h. The total cell lysates were detected by western blot analysis with specific antiserum. (B) *ΔhspL*(pTrc-HspL-His) and*ΔhspL*(pTrc-HspAT2-His) were grown in AB-MES (pH 5.5) containing 200 µM AS at 28°C with different concentrations of IPTG (0∼80 µM) for 24 h. The signal of VirB8 or sHsp-His was determined by western blot using anti-VirB8 or anti-His antiserium and the intensity of signal was quantified. The signal of VirB8 or sHsp-His was normalized by the signal of RpoA (loading control). The regression curve was created using the increase of signal intensity of VirB8 as Y-axis and sHsp-His as X-axis. The lineal range of signals was selected to calculate regression curve, the increase of signal intensity of VirB8 as Y-axis and sHsp-His as X-axis.

HspL is required for timely and optimal accumulation of several VirB proteins including VirB8 [25]. Thus, we next investigated whether the discrepancy between HspL and other sHsps in conferring VirB8 chaperone activity *in vitro* is reflected in their ability to maintain VirB8 protein levels *in vivo*. Thus, we expressed each of the four *shsp* genes encoding 6×His-tagged protein driven by the *trc* promoter in the *A. tumefaciens* Δ*hspL* mutant. This expression system allows the controlled expression of proteins by isopropylthio-β-galactoside (IPTG) induction and quantification of all four sHsps using an anti-His antibody. VirB8 and the sHsps were expressed abundantly in the presence of 200 µM AS and 0.4 mM IPTG for 24 hrs at 28°C (Figure 5A). HspL accumulated at slightly lower levels than the other sHsps based on the signals detected by the anti-His sera. Nonetheless, VirB8 accumulated at highest levels in the Δ*hspL* mutant with IPTG-induced His-tagged HspL, HspC, or HspAT2, whereas the VirB8 protein level did not increase in Δ*hspL* containing the vector alone or expressing HspAT1-His upon IPTG induction as compared to non-IPTG induction control (Figure 5A). As an internal control, RpoA accumulates at similar protein levels in the presence or absence of IPTG in all strains. Taken together, these data suggest that HspL functions as the most efficient molecular chaperone in maintaining VirB8 homeostasis, which may be critical for full virulence of *A. tumefaciens*.

To confirm the notion that lower amounts of HspL are sufficient to maintain the optimal VirB8 protein level, HspL-His and HspAT2-His were expressed at various levels and determined their effects on VirB8 accumulation in the Δ*hspL* background. VirB8 protein levels gradually increased in an IPTG concentration-dependent manner when cells were treated with AS and 2.5 to 80 µM of IPTG for 24 hr (Figure 5B). In the presence of identical IPTG concentrations, HspL always accumulated at lower amounts than HspAT2 (Figure 5B). By quantifying the relative amounts of VirB8 to HspL and HspAT2, respectively, it appeared that ∼3-fold more of HspAT2 than HspL was present in the cells (Figure 5B). This data suggested that HspL possesses higher activity than HspAT2 in maintaining VirB8 homeostasis in *A. tumefaciens* (*in vivo*), which is consistent with the chaperone activity observed *in vitro*.

### Chaperone Activity Analysis of HspL-HspAT2 Chimeric Proteins by Domain Swapping

**Figure 6:**
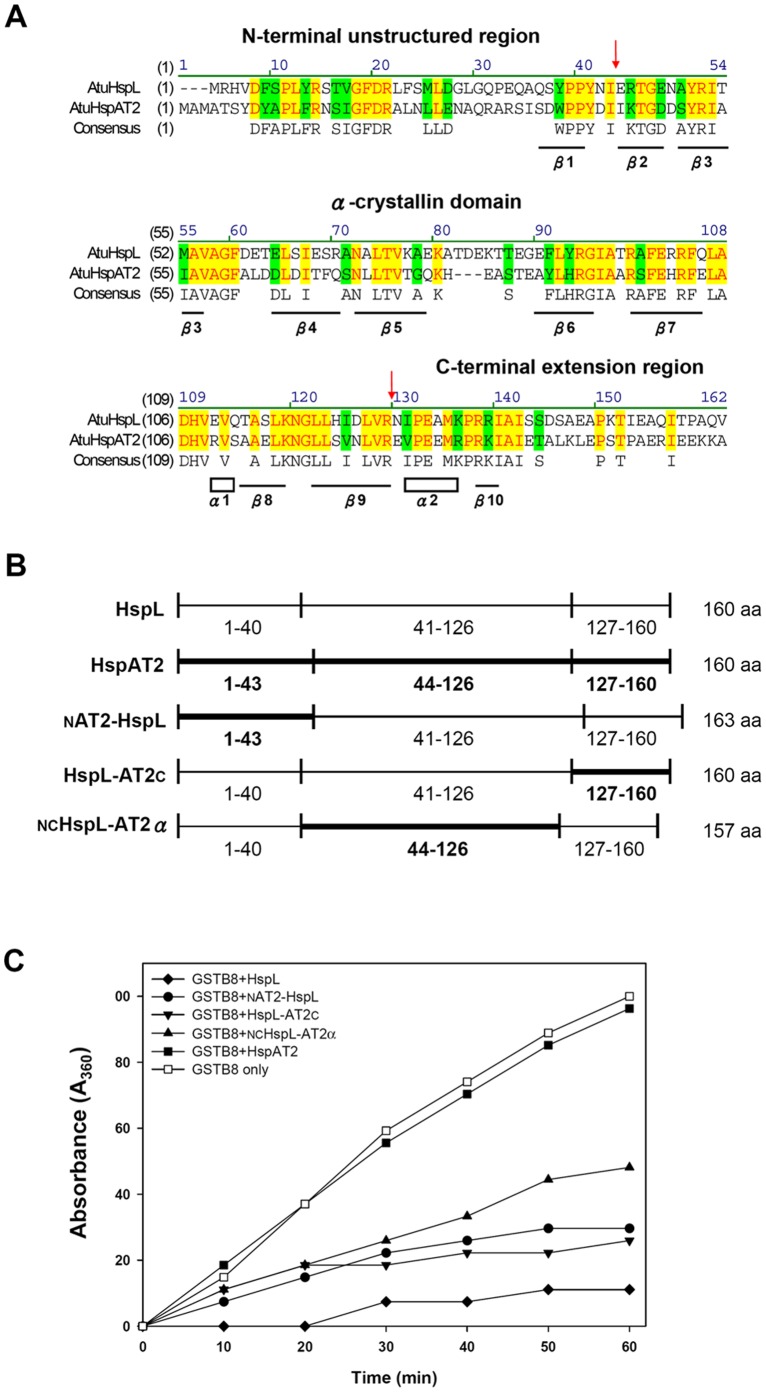
Chaperone activity assay of HspL/HspAT2 chimeric proteins using GST-VirB8 as the substrate. (A) Amino acid sequences of HspL and HspAT2 were aligned and the conserved amino acids are highlighted in yellow blocks and the blue and green blocks indicate the identical and similar amino acids, respectively. The boundary for N-terminal arm, α-crystallin domain, and C-terminal arm was indicated by arrows. (B) Diagram of the HspL/HspAT2 chimeric proteins. The HspL sequence is represented by thin lines, whereas HspAT2 is represented by thick lines with the respective position of amino acid residues indicated. The amino acid length of each protein is indicated to the right. (C) Thermal aggregation protection assays using GST-VirB8 (1 µM) was carried out at 50°C in the absence (□), or presence of HspL-His_6_ (♦), _N_AT2-HspL-His_6_ (•), HspL-AT2_C_-His_6_ (▾), _NC_HspL-AT2α-His_6_ (▴) and HspAT2–His_6_ (▪) at concentration of 2 µM (for GST-VirB8) or 1.2 µM (for CS) respectively. Aggregation was monitored in absorbance at 360 nm and presented as a function of time.

The data that HspL possesses higher activity than HspAT2 in protecting GST-VirB8 from aggregation *in vitro* and maintaining VirB8 homeostasis *in vivo* intrigued us to investigate the domain(s) of HspL in contributing higher chaperone activity than HspAT2. Amino acid sequence alignment of HspL and HspAT2 revealed 41.1% identity and 55.8% similarity (Fig. 6A). Among the three functional domains, the α-crystallin domain is most conserved (50.6% identity and 64.4% similarity), in contrast to more variations in the N-terminal arm (30.2% identity and 48.8% similarity) and C-terminal extension (32.4% identity and 44.1% similarity). To determine which domain(s) of HspL contribute the most to its chaperone activity towards VirB8, we generated a series of chimeric proteins by domain swapping between HspL and HspAT2. All chimeric proteins were expressed with a C-terminal His-tag and purified from *E. coli* to determine the chaperone activity in protecting GST-VirB8 from thermal aggregation *in vitro.* Interestingly, all chimeric proteins revealed chaperone activity higher than HspAT2 but lower than HspL (Figure 6C). However, the chimeric proteins _N_AT2-HspL and HspL-AT2_C_, which both retain the α-crystallin domain of HspL, exhibited slightly higher chaperone activity than _NC_HspL-AT2α (Figure 6C). The data suggested that each of the three domains of HspL contributes to efficiently protecting GST-VirB8 from aggregation. Among them, the α-crystallin domain of HspL may play the most critical role to confer higher activity than HspAT2 for its VirB8 chaperone activity.

## Discussion

The unusual presence of multiple sHsps in *Rhizobiaceae* presumably helps to protect cells under various stresses. However, it remains unclear whether each of the sHsp genes encoded in the same genome share redundant or distinct functions in specific biological process. In this study, we explored the biochemical and biological functions of the *Agrobacterium* four sHsps in their involvement in T4SS-mediated DNA transfer and tumorigenesis and provided the molecular basis explaining the dominant role of HspL in promoting T4SS functions.

Except for HspL, the precise roles of the sHsps in *A. tumefaciens* remain unknown. The fact that single deletions of *hspC, hspAT1,* or *hspAT2* did not affect T4SS-mediated DNA transfer and tumorigenesis is consistent with the low level expression of these genes in wild type *A. tumefaciens* at both mRNA and protein levels (Figure 1 and 2). These data suggested that HspL is the dominant sHsp responsible for *Agrobacterium* virulence. Interestingly, the significantly lower T4SS-mediated DNA transfer and tumorigenesis in the triple (Δ*L,C*, *AT2*) and quadruple mutants (Δ*L,C*, *AT1, AT2* or Δ*4shsp*) as compared to single *hspL* deletion mutant (Δ*hspL*) (Figure 1) suggested that HspC, HspAT1, or HspAT2 can play a minor role in T4SS functioning. This could be explained by the low abundance but significant AS-induced expression of *hspC, hspAT1,* and *hspAT2* at both mRNA and protein levels (Figure 2). Their expression could exert a role in partially compensating for HspL functions in promoting T4SS in the absence of *hspL*. The complementation of the Δ*4sHsps* phenotype by the overexpression of each of sHsps (Figure 3) indeed suggested that HspC, HspAT1, and HspAT2 could substitute the biochemical functions of HspL when they are properly expressed. This argument is further supported by their principal chaperone activity in protecting thermolabile protein CS from aggregation *in vitro* (Figure 4A).

The native promoters used for the expression of HA-tagged sHsps on plasmid are indeed functional because all four sHsp genes are responsive to AS with protein accumulation patterns generally similar to that of mRNA (Figure 2B). HspL-HA, HspAT1-HA, and HspAT2-HA but not HspC-HA are also heat-inducible (data not shown), which are consistent with previous studies on the heat responsiveness of *hspL, hspAT1,* and *hspAT2* but not *hspC* at mRNA levels [23]. However, while we consistently observed slightly higher AS-induction for *hspAT1* mRNA in the Δ*hspL* mutant as compared to that in WT (Figure 2A), HspAT1-HA protein levels accumulated at lower levels in Δ*hspL* (Figure 2B). Because several VirB proteins accumulate at lower levels in the absence of *hspL* [25] and both chaperone and its interacting substrate become stabilized when they interact with each other [1], [27], we propose that HspAT1 (and maybe other sHsps) protein abundance may be also regulated at the protein level by stabilization when binding to the substrate. Future work to identify the substrates of HspAT1 and other sHsps shall advance our understanding how HspC, HspAT1, and HspAT2 contribute in promoting T4SS functions.

At present, we do not exclude the possibility that the reduced T4SS-mediated DNA transfer and virulence detected in the triple (Δ*L,C*, *AT2*) and quadruple mutants (Δ*L,C*,*AT1, AT2* or Δ*4sHsps*) are based on other functions than direct impact on the T4SS. It is reasonable to assume that *hspC, hspAT1,* and *hspAT2* function to protect agrobacterial cells in specific stress environments, which are normally not critical for *Agrobacterium* virulence. However, their impacts in virulence became detectable when HspL is no longer around to maintain normal T4SS functions. Thus, future work to compare the transcriptomes and proteomes among wild type and various mutants shall shed light to understand the mechanisms.

While all four sHsps exhibit comparable chaperone activity in protecting CS from thermal aggregation, only HspL is capable in protecting GST-VirB8 from aggregation when sHsp/substrate molar ratio at 2∶1 (Figure 4A and 4B). Further kinetic analysis for HspAT2 demonstrated that HspAT2 is also capable to function as a VirB8 chaperone but requires higher sHsp/substrate molar ratio (Figure 4C). These biochemical activities *in vitro* are fully consistent with the *in vivo* studies, in which lower amounts of HspL than HspAT2 were sufficient to maintain VirB8 homeostasis in *A. tumefaciens* (Figure 5). Interestingly, the lack or lower chaperone activity of HspC, HspAT1, and HspAT2 as compared to HspL for VirB8 is not due to their inability to recognize the substrate because they are able to interact with GST-VirB8 by co-purification assay in *E. coli* (Figure S1). Thus, finding the native substrates of sHsp could be challenging because the identified interacting proteins may not be necessarily the clients or substrates. To date, little is known about the substrate specificity of sHsps *in vivo* [5]. However, the data from yeast and bacteria suggest a general role of sHsp in protecting a wide range of proteins in the cells [28], [29]. Thus, while VirB8 is the only native substrate of HspL identified in *A. tumefaciens* [26], it is possible that HspL may also protect other highly expressed Vir proteins under virulence-induced growth environment. Several VirB proteins accumulating at lower levels in the absence of HspL [25] could be the potential targets for future studies.

All three domains (variable N-terminal region, central conserved α-crystallin domain, C-terminal extension) of sHsp play critical roles in conferring chaperone activity [1]. Our domain swapping experiments for HspL and HspAT2 revealed that each of the three domains of HspL is important for its optimal chaperone activity for VirB8 (Figure 6). Interestingly, our data suggest that the chimeric protein containing the α-crystallin domain of HspL flanked by both N- and C-termininal domains of HspAT2 exerts significantly higher VirB8 chaperone activity than that of HspAT2 (Figure 6). Although the α-crystallin domain is highly conserved between HspL and HspAT2, we noticed that the linker region between β-5 and β-6 is variable (Figure 6A). Thus, future studies to fine map the motif contributing to higher VirB8 chaperone activity for HspL and determine the effects in dimer and oligomer formation may provide new insights to advance our knowledge on the substrate selection of sHsps.

Most studies of sHsps focus on their structure and biochemical property and mechanisms, whereas little is known about their biological functions *in vivo* [1]. Specific roles of multiple sHsp genes in many rhizobia are so far only speculated. In this study, we provided compelling evidence showing that HspL is the key sHsp responsible for promoting T4SS-mediated DNA transfer and virulence in *A. tumefaciens* and other three sHsps could also play minor roles but their involvements are only evident in the absence of HspL. HspL is the dominant sHsp in T4SS functioning not only because it is highly induced in in virulence-induced condition but also because it is a more efficient VirB8 chaperone over other sHsps. It would be interesting to future explore whether the involvement of sHsp in T4SS functions is a general theme in bacteria containing T4SSs.

## Materials and Methods

### Plasmids, Bacterial Strains, and Growth Conditions

The information for the bacterial strains, plasmids, and primers used in this study were summarized in Information S1, Table S1, and Table S2. *A. tumefaciens* strains were routinely grown in 523 medium at 28°C [30] and *E. coli* strains were grown in Luria-Bertani medium at 37°C [31]. The final concentrations of antibiotics added in the cultures were: ampicillin (Ap, 100 µg/ml) or kanamycin (Km, 50 µg/ml) for *E. coli* strains; erythromycin (Em, 50 µg/ml), kanamycin (Km, 50 µg/ml), rifampicin (Rm, 50 µg/ml), or spectinomycin (Sp, 250 µg/ml) for *A. tumefaciens* strains. The induction of *A. tumefaciens* virulence genes was performed as described before [25]. In brief, the 525-overnight grown cells were sub-cultured with OD_600 nm_ = 0.1 in I-medium (AB-MES, pH 5.5) containing 200 µM acetosyringone (AS; Sigma-Aldrich, St. Louis, MO, USA) and grown in indicated temperature and time period in the absence of antibiotics. To induce the expression of sHsps from pTrc200, IPTG was added with desired concentration in I-medium containing 200 µM AS for growth as indicated temperature and time period.

### Virulence Assay

Quantitative tumorigenesis assays with potato tuber discs and the conjugation assay were performed as described [25] unless specified. Potato tuber discs were infected at 10^8^ cells/ml and the tumorigenesis efficiency is presented as number of tumor per disc, with standard errors averaged from the results of 60 potato discs for each strain. All *A. tumefaciens* strains harboring the IncQ plasmid RSF1010 derivative pML122*Δkm*::Tc^R^ served as the donor strains and co-incubated with the recipient strain NT1RE-Sp on the I-medium agar in the presence of 200 µM AS at 25°C for 3 days. The transfer efficiency of RSF1010 was evaluated as number of transconjugants per input donor. Average values for absolute and relative RSF1010 transfer efficiency from three biological repeats are shown with standard deviation, in which the efficiency of wild-type strain NT1RE(pJK270) was set at 100% and that of other strains is shown relative to that of NT1RE(pJK270).

### Quantitative Real-time PCR (qRT-PCR)

Total RNA from *A. tumefaciens* strains was extracted by the hot-phenol method [32] and treated with DNase I (Promega) to eliminate DNA contamination. The method for qRT-PCR was carried out as described [33] using Power SYBR Green PCR Master Mix reagent (Applied Biosystems) and the ABI 7500 Real-Time PCR System (Applied Biosystems). The program for qRT-PCR was 2 min at 50°C, 10 min at 95°C, 40 cycles of 15 s at 95°C/1 min at 60°C. Expression was normalized to that of 16S rRNA as an internal control by the 2^−ΔΔCt^ method [34].

### Overexpression of His-tagged sHsp and Glutathione-S-transferase (GST)-VirB8 in *E. coli*

The expression vectors pET22b(+) and pET42b(+) were used to construct the plasmids for overexpression His-tagged small heat shock proteins (HspL, −C, −AT1, −AT2, HspL truncate proteins and chimeric proteins) and VirB8 fused with GST respectively. These proteins were overexpressed in *E. coli* BL21(DE3) by IPTG (0.4 mM) induction at 37°C for 1.5 h (in the case of HspL-His) or 28°C for 2 h (GST-VirB8) according to the instructions of the pET user manual (Novagen, EMD Biosciences, Inc, Germany).

### Purification of Recombinant His-tagged sHsps and GST-VirB8 Proteins

The purification of the recombinant proteins was performed as described [26].

### SDS-PAGE and Western Blot Analysis

Proteins were resolved by either Glycine-SDS-PAGE [31] or Tricine-SDS-PAGE [35]. Western blot analysis was performed as described previously [36] using primary polyclonal antibody against VirB8, RpoA, or 6xHis, followed by secondary antibody using horseradish peroxidase - conjugated goat anti-rabbit and detection using the Western Lightning System (Perkin Elmer, Boston, MA). Chemiluminescent signals were visualized on a High performance chemiluminescence film (GE Healthcare) or used BioSpectrumAC Imaging System (Ultra-Violet Products Ltd., UK) to detect and calculate the photon intensity of signals.

### Thermal Aggregation Protection Assay

Thermal aggregation protection assays were carried out as described [26]. The artificial model substrate citrate synthase (CS, Sigma-Aldrich) or native client VirB8 fused with GST were used as substrates. CS was dialyzed against 50 mM sodium phosphate buffer (pH8.0) and stored at −20°C before use. CS and GST-VirB8 were incubated in the presence or absence of His-tagged sHsp by heat treatment in the reaction buffer (50 mM sodium phosphate pH 8.0, 150 mM NaCl) at 43°C and 50°C, respectively. Chaperone activity is assessed by the reduced level of substrate aggregation measured by light scattering at A_360_.

## Supporting Information

Figure S1 **The interaction of GST-VirB8 and sHsps.** *E. coli* BL21(DE3) strain containing both pETGSTB8 (for expression of GST-VirB8) and pETHspL, pETHspC, pETHspAT1 or pETHspAT2 (for expression of each sHsp) respectively was induced by 0.4 mM IPTG at 28°C for 2 h. The total soluble protein extracts (T) were run through Glutathione-agarose (G) to purify GST-VirB8 and its interacting proteins. The eluates were analyzed by western blot with anti-His antiserum to visualize each sHsp.
(PDF)

Table S1 **Bacterial strains and plasmids.**
(PDF)

Table S2 **Primer information.**
(PDF)

Information S1 (PDF)
