# Supplementary material for: One out of Four: HspL but No Other Small Heat Shock Protein of Agrobacterium tumefaciens Acts as Efficient Virulence-Promoting VirB8 Chaperone
Source: PLoS One. 2012 Nov 21;7(11):e49685. doi: 10.1371/journal.pone.0049685 (PMC3504140; doi:10.1371/journal.pone.0049685)
Supplement: Figure S1 — The interaction of GST-VirB8 and sHsps. E. coli BL21(DE3) strain containing both pETGSTB8 (for expression of GST-VirB8) and pETHspL, pETHspC, pETHspAT1 or pETHspAT2 (for expression of each sHsp) respectively was induced by 0.4 mM IPTG at 28°C for 2 h. The total soluble protein extracts (T) were run through Glutathione-agarose (G) to purify GST-VirB8 and its interacting proteins. The eluates were analyzed by western blot with anti-His antiserum to visualize each sHsp. (PDF) [file pone.0049685.s001.pdf]

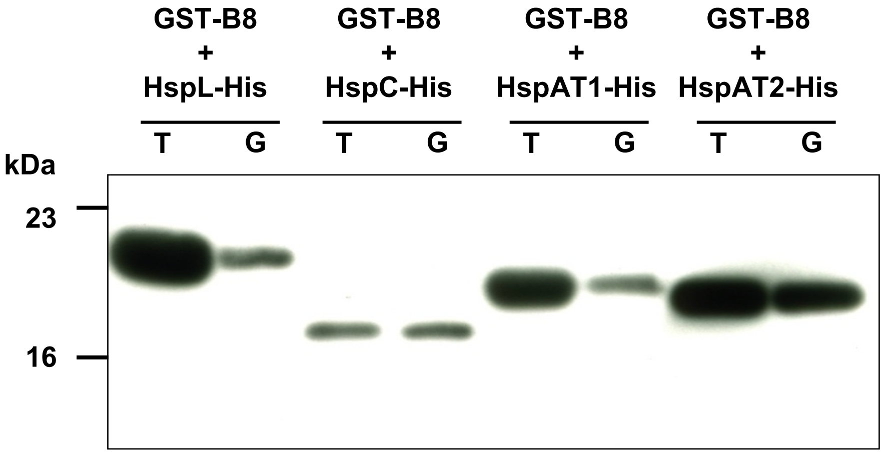

FIGURE S1. The interaction of GST-VirB8 and sHsps. *E. coli* BL21(DE3) strain containing both pETGSTB8 (for expression of GST-VirB8) and pETHspL, pETHspC, pETHspAT1 or pETHspAT2 (for expression of each sHsp) respectively was induced by 0.4 mM IPTG at 28°C for 2 h. The total soluble protein extracts (T) were run through Glutathione-agarose (G) to purify GST-VirB8 and its interacting proteins. The eluates were analyzed by western blot with anti-His antiserum to visualize each sHsp.

REFERENCES

1. **Kao, J. C., K. L. Perry, and C. I. Kado.** 1982. Indoleacetic acid complementation and its relation to host range specifying genes on the Ti plasmid of *Agrobacterium tumefaciens*. Mol Gen Genet **188:**425-32.
2. **Ma, L. S., F. Narberhaus, and E. M. Lai.** 2012. IcmF Family Protein TssM Exhibits ATPase Activity and Energizes Type VI Secretion. J Biol Chem **287:**15610-21.
3. **Sambrook, J., and D. Russell.** 2001. Molecular cloning: a laboratory manual, Third ed. Cold Spring Harbor Laboratory Press, Cold Spring Harbor, New York.
4. **Schmidt-Eisenlohr, H., N. Domke, and C. Baron.** 1999. TraC of IncN plasmid pKM101 associates with membranes and extracellular high-molecular-weight structures in *Escherichia coli*. J Bacteriol **181:**5563-5571.
5. **Studier, F. W., A. H. Rosenberg, J. J. Dunn, and J. W. Dubendorff.** 1990. Use of T7 RNA polymerase to direct expression of cloned genes. Methods Enzymol **185:**60-89.
6. **Tsai, Y. L., Y. R. Chiang, F. Narberhaus, C. Baron, and E. M. Lai.** 2010. The small heat-shock protein HspL Is a VirB8 chaperone promoting type IV secretion-mediated DNA transfer. J Biol Chem **285:**19757-66.
7. **Tsai, Y. L., M. H. Wang, C. Gao, S. Klusener, C. Baron, F. Narberhaus, and E. M. Lai.** 2009. Small heat-shock protein HspL is induced by VirB protein(s) and promotes VirB/D4-mediated DNA transfer in *Agrobacterium tumefaciens*. Microbiology **155:**3270-80.
