# Supplementary material for: One out of Four: HspL but No Other Small Heat Shock Protein of Agrobacterium tumefaciens Acts as Efficient Virulence-Promoting VirB8 Chaperone
Source: PLoS One. 2012 Nov 21;7(11):e49685. doi: 10.1371/journal.pone.0049685 (PMC3504140; doi:10.1371/journal.pone.0049685)
Supplement: Table S1 — Bacterial strains and plasmids. (PDF) [file pone.0049685.s002.pdf]

Table S1. Bacterial strains and plasmids

Strains/plasmids	Relevant characteristics	References/ sources
A. tumefaciens		
NT1RE(pJK270)	Rm^R, Em^R, Km^R/Nm^R, *Agrobacterium tumefaciens* strain NT1RE containing pJK270, which is pTiC58Tra^C with *Tn5* insertion in T-DNA region without affecting virulence	[1]
NT1RE-Sp	Rm^R, Em^R, Sp^R, NT1RE containing spectinomycin resistant gene (*aadA*)	[2]
Δ*hspL*	Rm^R, Em^R, Km^R/Nm^R, *hspL* deletion mutant in NT1RE(pJK270)	[2]
ΔhspC	Rm^R, Em^R, Km^R/Nm^R, *hspC* deletion mutant in NT1RE(pJK270)	This study
ΔhspAT1	Rm^R, Em^R, Km^R/Nm^R, *hspAT1* deletion mutant in NT1RE(pJK270)	This study
ΔhspAT2	Rm^R, Em^R, Km^R/Nm^R, *hspAT2* deletion mutant in NT1RE(pJK270)	This study
ΔhspL, *C*	Rm^R, Em^R, Km^R/Nm^R, *hspL hspC* double-deletion mutant in NT1RE(pJK270)	This study
ΔhspL, *AT2*	Rm^R, Em^R, Km^R/Nm^R, *hspL hspAT2* double-deletion mutant in NT1RE(pJK270)	This study
ΔhspC, *AT2*	Rm^R, Em^R, Km^R/Nm^R, *hspC hspAT2* double-deletion mutant in NT1RE(pJK270)	This study
ΔhspL, *C*, *AT2*	Rm^R, Em^R, Km^R/Nm^R, *hspL hspC hspAT2* triple-deletion mutant in NT1RE(pJK270)	This study
Δ*4sHsps*	Rm^R, Em^R, Km^R/Nm^R, *hspL hspC hspAT1 hspAT2* quadruple-deletion mutant in NT1RE(pJK270)	This study
E. coli		
DH10B	Host for DNA cloning	Invitrogen
BL21(DE3)	Host for overexpressing proteins driven by T7 promoter	[3]
Plamid		
pJQHspC	Gm^R, for *hspC* deletion construct	This study
pJQHspAT1	Gm^R, for *hspAT1* deletion construct	This study
pJQHspAT2	Gm^R, for *hspAT2* deletion construct	This study
pETHspL	Ap^R, overexpression of HspL-His in *E. coli*	[2]
pETHspC	Ap^R, overexpression of HspC-His in *E. coli*	This study

pETHspAT1	Ap^R, overexpression of HspAT1-His in *E. coli*	This study
pETHspAT2	Ap^R, overexpression of HspAT2-His in *E. coli*	This study
pETGSTB8	Km^R, overexpression of GST-VirB8 in *E. coli*	[4]
$pET_{N}AT2$-HspL	Ap^R, overexpression of $_{N}AT2$-HspL-His chimeric protein, in *E. coli*	This study
pETHspL-$AT2_{C}$	Ap^R, overexpression of HspL-$AT2_{C}$-His chimeric protein, in *E. coli*	This study
pET_{NC}HspL-AT2α	Ap^R, overexpression of $_{NC}$HspL-AT2α-His chimeric protein, in *E. coli*	This study
pN-HspL-HA	Sp^R, expression of HspL-HA by native promoter	This study
pN-HspC-HA	Sp^R, expression of HspC-HA by native promoter	This study
pN-HspAT1-HA	Sp^R, expression of HspAT1-HA by native promoter	This study
pN-HspAT2-HA	Sp^R, expression of HspAT2-HA by native promoter	This study
pTrc200	Sp^R, pVS1 origin *lacI*q, *tac* promoter expression vector	[5]
pTrc200HA	Sp^R, HA sequence inserted between *Pst*I and *Hind*III site of pTrc200	[6]
pTrcHspL-His	Sp^R, expression of HspL-His by *tac* promoter	This study
pTrcHspC-His	Sp^R, expression of HspC-His by *tac* promoter	This study
pTrcHspAT1-His	Sp^R, expression of HspAT1-His by *tac* promoter	This study
pTrcHspAT2-His	Sp^R, expression of HspAT2-His by *tac* promoter	This study

REFERENCES

1. Kao JC, Perry KL, Kado CI (1982) Indoleacetic acid complementation and its relation to host range specifying genes on the Ti plasmid of Agrobacterium tumefaciens. Mol Gen Genet 188: 425-432.
2. Tsai YL, Wang MH, Gao C, Klusener S, Baron C, et al. (2009) Small heat-shock protein HspL is induced by VirB protein(s) and promotes VirB/D4-mediated DNA transfer in Agrobacterium tumefaciens. Microbiology 155: 3270-3280.
3. Studier FW, Rosenberg AH, Dunn JJ, Dubendorff JW (1990) Use of T7 RNA polymerase to direct expression of cloned genes. Methods Enzymol 185: 60-89.
4. Tsai YL, Chiang YR, Narberhaus F, Baron C, Lai EM (2010) The small heat-shock protein HspL is a VirB8 chaperone promoting type IV secretion-mediated DNA transfer. J Biol Chem 285: 19757-19766.
5. Schmidt-Eisenlohr H, Domke N, Baron C (1999) TraC of IncN plasmid pKM101 associates with membranes and extracellular high-molecular-weight structures in Escherichia coli. Journal of Bacteriology 181: 5563-5571.
6. Ma LS, Narberhaus F, Lai EM (2012) IcmF family protein TssM exhibits ATPase activity and energizes type VI secretion. J Biol Chem 287: 15610-15621.
