# Supplementary material for: One out of Four: HspL but No Other Small Heat Shock Protein of Agrobacterium tumefaciens Acts as Efficient Virulence-Promoting VirB8 Chaperone
Source: PLoS One. 2012 Nov 21;7(11):e49685. doi: 10.1371/journal.pone.0049685 (PMC3504140; doi:10.1371/journal.pone.0049685)
Supplement: Table S2 — Primer information. (PDF) [file pone.0049685.s003.pdf]

Table S2. Primer information

Name and size of PCR product	Primers	Sequence[a]
hspC for pET22b(+)	hspC-F-EN	5'- GAATTC**CATATG**AGCCGCATGACGCCATT -3'
	hspC-R-XhoI	5'- **CTCGAG**CTCTGAGACGGAAATAT -3'
hspAT1 for pET22b(+)	hspAT1-F-EN	5'-GAATTC**CATATG**GCAACATCTTACGACTA-3'
	hspAT1-R-XhoI	5'-**CTCGAG**AGCGGCCTTCTGCGCTT-3'
hspAT2 for pET22b(+)	hspAT2-F-EN	5'-GAATTC**CATATG**GCTATGGCAACATCCTA-3'
	hspAT2-R-XhoI	5'-**CTCGAG**TGCAGCCTTCTTTTCTT-3'
hspL-alpha-C for chimeric construct	L-K-alpha-F	5'-CCCTATGACATCGAACGAACAGGTGA-3'
	hspL-XhoI-R	5'-**CTCGAG**GTTGACCTGAGCCGGG-3'
hspL-N-alpha for chimeric construct	hspL-NdeI-F	5'-**CATATG**CGTCACGTTGATTTTTCCC-3'
	L-K-alpha-R	5'-TCTTCCGGAACCTCGCGGACGAGGTCG-3'
hspAT2-N for chimeric construct	hspAT2-F-EN	5'-GAATTC**CATATG**GCTATGGCAACATCCTA-3'
	AT2-NK-R	5'-TCACCTGTTCGTTCGATGTCATAGGG-3'
hspAT2-alpha for chimeric construct	AT2-K-alpha-F	5'-CCCTATAATATCATCAAGACCGGCGA-3'
	AT2-K-alpha-R	5'-GCTTCCGGAATATTGCGAACGAGAT-3'
hspAT2-C for chimeric construct	AT2-CK-F	5'-CGACCTCGTCCGCGAGGTTCCGGAAGA-3'
	hspAT2-R-XhoI	5'-**CTCGAG**TGCAGCCTTCTTTTCTT-3'
hspL promoter＋*hspL* (no stop codon)	Compl-hspL-FP	5'-**GGATCCAAGCTT**TGTCTTCACTGGCGCAAT-3'
	Compl-hspL-RP	5'-**CTGCAG**GTTGACCTGAGCCGGGGTA-3'
hspC promoter＋*hspC* (no stop code)	Compl-hspC-FP	5'-**GGATCCAAGCTT**AGGACTTCCAAAGGCGAGCA-3'
	Compl-hspC-RP	5'-**CTGCAG**CTCTGAGACGGAAATATTAAT-3'
hspAT1 promoter＋*hspAT1* (no stop codon)	Compl-hspAT1-FP	5'-**GGATCCAAGCTT**AGAAGACGCTCGACACGTTG-3'
	Compl-hspAT1-RP3	5'-CGC**CTGCAG**AGCGGCCTTCTGCGCTTC-3'
hspAT2 promoter＋*hspAT2* (no stop codon)	Compl-hspAT2-FP	5'-**GGATCCAAGCTT**CTTCGAGCGCGAAGAAGAAG-3'
	Compl-hspAT2-RP	5'-**CTGCAG**TGCAGCCTTCTTTTCTTCGAT-3'
	His-PstI-R	5'- **CTGCAG**TTATCAGTGGTGGTGGTGGTGGTG-3'
For *hspC* deletion construct	hspC-FP1-1	5'-**TCTAGA**ATTCCAGCATTTCCG-3'
	hspC-RP1-1	5'-**GGATCC**CGCTTCCGGCAACCT-3'
	hspC-FP2-1	5'-**GGATCC**ATGAACGCTGGAATC-3'
	hspC-RP2-1	5'-**ACTAGT**GGCGGCTTGGGAATG-3'
For *hspAT1* deletion construct	hspAT1-FP1	5'-**TCTAGA**CCGAGCTGTCCGTCCGCCGA-3'
	hspAT1-RP1	5'-**GGATCC**AGCCCCGTCGTCAGCGGCT-3'
	hspAT1-FP2	5'-**GGATCC**GGGTTGGGAAGGAGAGTATC-3'
	hspAT1-RP2	5'-**CTGCAG**CCTGAAGCTCATCGATGGA-3'

For *hspAT2* deletion construct	hspAT2-FP1-1	5'-**TCTAGA**CCCACCTTGGTCGCGATG-3'
	hspAT2-RP1-2	5'-**GGATCC**TTGAAGCAACATGGGTAC-3'
	hspAT2-FP2-3	5'-**AGATCT**GCGACGCCCGTCAACATTG-3'
	hspAT2-RP2	5'-**ACTAGT**CGTGTAAAAGTCTCGCGCCA-3'

[a]The sequences of designed restriction enzyme sites were shown in bold and the sequences complementary to specific region in *A. tumefaciens* genome is shown in Italian
