# Supplementary material for: One out of Four: HspL but No Other Small Heat Shock Protein of Agrobacterium tumefaciens Acts as Efficient Virulence-Promoting VirB8 Chaperone
Source: PLoS One. 2012 Nov 21;7(11):e49685. doi: 10.1371/journal.pone.0049685 (PMC3504140; doi:10.1371/journal.pone.0049685)
Supplement: Information S1 — (PDF) [file pone.0049685.s004.pdf]

Supporting Information S1

Plasmid construction and generation of the sHsp mutants

The information of bacterial strains, plasmids, and primers used in this study is listed in Table S1 and Table S2. The techniques used for DNA cloning and PCR were performed by standard protocols [1]. Plasmid DNA isolation was performed using Plasmid Miniprep Purification Kit provided by GeneMark (Taipei, Taiwan).

All markerless deletion mutants were generated in *A. tumefaciens* NT1RE(pJK270) via double crossover with the suicide plasmid pJQ200KS as described previously (7). The plasmids pJQHspC, pJQhspAT1, and pJQHspAT2 were created for *hspC*, *hspAT1* and *hspAT2* gene deletion, respectively. The primers hspC-FP1-1 and hspC-RP1-1 were used to amplify the upstream fragment of *hspC* gene; hspC-FP2-1 and hspC-RP2-1 were for *hspC* downstream fragment amplification; hspAT1-FP1 and hspAT1-RP1 were for *hspAT1* upstream fragment amplification; hspAT1-FP2 and hspAT1-RP2 were for *hspAT1* downstream fragment amplification; hspAT2-FP1-1 and hspAT2-RP1-2 were for *hspAT2* upstream fragment amplification; hspAT2-FP2-3 and hspAT2-RP2 were for *hspAT2* downstream fragment amplification.

For the expression of His-tagged sHsp proteins, the DNA fragment containing *hspC, hspAT1,* or *hspAT* gene ORF without the stop codon was amplified by PCR with specific primers and the amplified DNA fragment was digested with *Nde*I and *Xho*I, and inserted at the same site of pET-22b(+). For the expression of HA-tagged sHsp proteins driven by native promoter, the DNA fragment containing *hspC, hspAT1,*or *hspAT* promoter and ORF without the stop codon was amplified by PCR with specific primers. The amplified DNA fragment was digested with *Bam*HI and *Pst*I, and inserted at the same site of pTrc200HA. For the expression of *hspL* driven by *trc* promoter, the DNA fragment containing *hspL* gene was amplified by primers

T7P and His-PstI-R using pETHspL as a template and then digested with *Xba*I/*Pst*I and ligated into pTrc200 at the same sites to result in plasmid pTrcHspL-His. For the expression of *hspC*, *hspAT1,* and *hspAT* driven by *trc* promoter, the DNA fragment containing the *hspC*, *hspAT1,* and *hspAT* was digested with *Xba*I/*Xho*I from pETHspC, pETHspAT1, and pETHspAT2, respectively and ligated into pTrcHspL-His at the same site. For the expression of chimeric proteins, two PCR products were amplified as follows. PCR product AT2-N was amplified by primers HspAT2-F-EN and AT2-NK-R using pETHspAT2 as a template. PCR product L-alpha-C was amplified by primers L-K-alpha-F and hspL-XhoI-R using pETHspL as a template. Both PCR products were mixed for extension by PCR using HspAT2-F-EN and hspL-XhoI-R as primers. The amplified DNA fragment was digested and cloned into pET-22b(+) at *Nde*I/*Xho*I sites to result in plasmid pET_NAT2-HspL. Accordingly, pETHspL-$AT2_C$ was cloned with the same way from two PCR products LN-alpha (hspL-NdeI-F and L-K-alpha-R as primers) and AT2-c (AT2-CK-F and hspAT2-R-XhoI as primers); pET_{NC}HspL-AT2α from L-N (HspD-NdeI-F and L-NK-R as primers), AT2-alpha (AT2-K-alpha-F and AT2-K-alpha-R as primers), and L-C (HspL-CK-F and HspD-XhoI-R as primers). The obtained plasmid constructs were confirmed by restriction mapping and DNA sequencing.

REFERENCES

1. Sambrook J, Russell D (2001) Molecular cloning: a laboratory manual. Cold Spring Harbor, New York: Cold Spring Harbor Laboratory Press.
